# The genome sequence of the clouded yellow, *Colias crocea* (Geoffroy, 1785)

**DOI:** 10.12688/wellcomeopenres.17292.1

**Published:** 2021-10-22

**Authors:** Sam Ebdon, Alex Mackintosh, Alex Hayward, Karl Wotton, Darwin Tree of Life Barcoding collective, Wellcome Sanger Institute Tree of Life programme, Wellcome Sanger Institute Scientific Operations: DNA Pipelines collective, Tree of Life Core Informatics collective, Darwin Tree of Life Consortium

**Affiliations:** 1 Institute of Evolutionary Biology, University of Edinburgh, Edinburgh, UK; 2 University of Exeter, Penryn, UK

**Keywords:** Colias crocea, Colias croceus, clouded yellow, genome sequence, chromosomal

## Abstract

We present a genome assembly from an individual female *Colias crocea *(also known as *Colias croceus*; the clouded yellow; Arthropoda; Insecta; Lepidoptera; Pieridae). The genome sequence is 325 megabases in span. The complete assembly is scaffolded into 32 chromosomal pseudomolecules, with the W and Z sex chromosome assembled. Gene annotation of this assembly on Ensembl has identified 13,803 protein coding genes.

## Species taxonomy

Eukaryota; Metazoa; Ecdysozoa; Arthropoda; Hexapoda; Insecta; Pterygota; Neoptera; Endopterygota; Lepidoptera; Glossata; Ditrysia; Papilionoidea; Pieridae; Coliadinae; Colias; *Colias crocea* (also known as *Colias croceus*) (Geoffroy, 1785) (NCBI:txid72248).

## Background

*Colias crocea* (or *croceus*), the clouded yellow, is a butterfly found in Europe, the middle east, and north Africa. This continuously-brooded migratory species visits the UK in the end of spring and summer, supplementing a small breeding population in the south. The larvae feed on a wide variety of leguminous plants, such as clovers ( *Trifolium* sp.), alfalfa ( *Medicago sativa*) and vetches ( *Vicia* sp.). Despite recent declines, *C. crocea* has seen a large increase in both abundance and occurrence in the last 50 years in the British Isles ( Fox *et al*., 2015) and is listed as Least Concern in the IUCN Red List (Europe) ( van Swaay *et al*., 2010). A white polymorphism known as Alba (form *helice*) is associated with an alternative life-history strategy, where females reallocate wing pigment resources to somatic and reproductive development. This is associated with the insertion of a transposable element downstream of the homeobox transcription factor *BarH-1* ( Woronik *et al*., 2019). *Colias crocea* has 31 pairs of chromosomes, a genome size of approximately 318.6 Mb ( Woronik *et al*., 2019), and is female heterogametic (WZ). We note the recent production of a high-quality genome assembly for *C. crocea* ( Woronik *et al*., 2019), and believe the sequence described here, generated as part of the Darwin Tree of Life project, will further aid understanding of the biology and ecology of this butterfly.

## Genome sequence report

**Figure 1.**
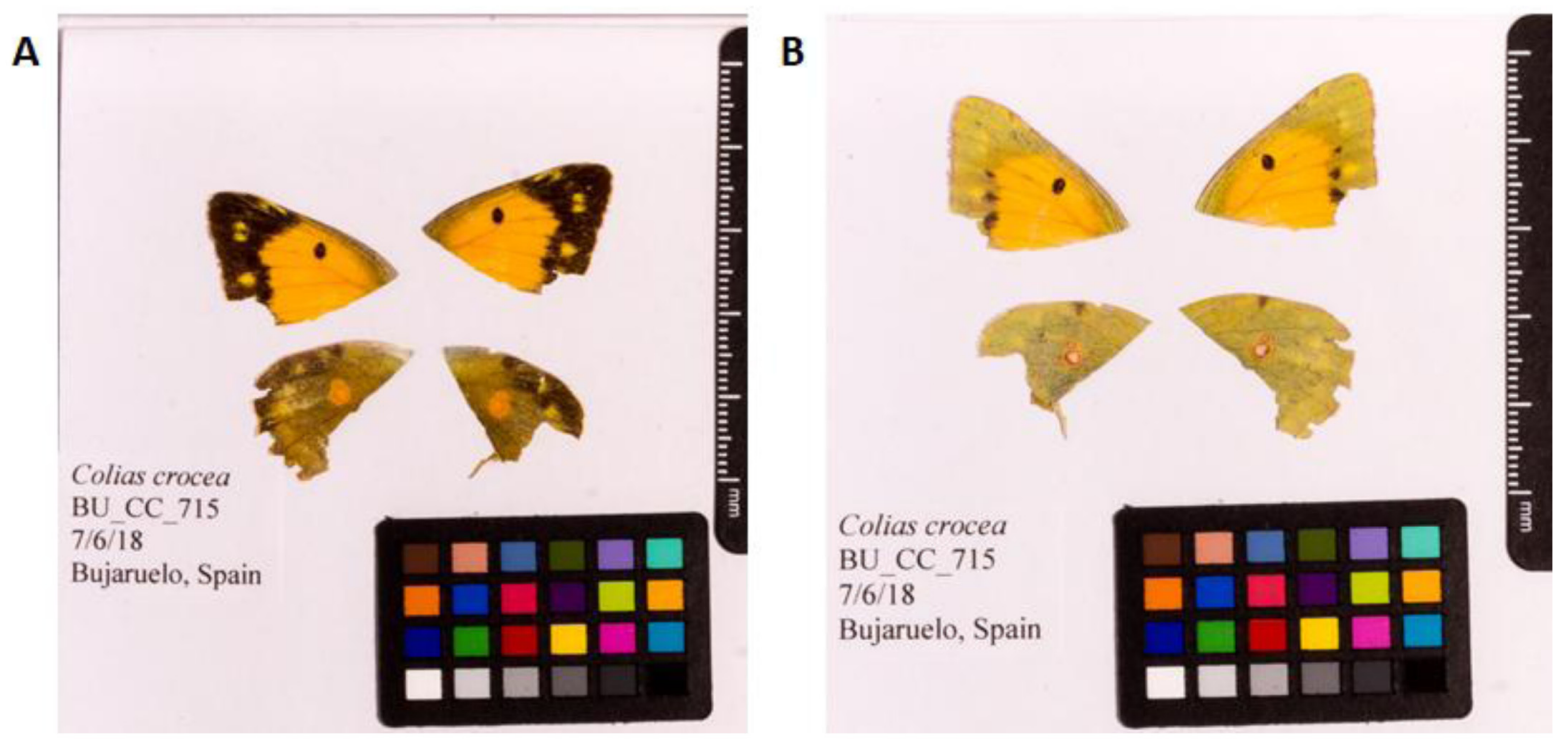
Fore and hind wings of *Colias crocea* specimen from which the genome was sequenced. ( **A**) Dorsal surface view of wings from specimen BU_CC_715 (ilColCroc2) from Bujaruelo, Spain, used to generate Pacific Biosciences and 10X genomics data. ( **B**) Ventral surface view of wings from specimen BU_CC_715 (ilColCroc2) from Bujaruelo, Spain, used to generate Pacific Biosciences and 10X genomics data.

The genome was sequenced from a single female *C. crocea* collected from Bujaruelo, Aragon, Spain (latitude 42.7, longitude -0.1) ( Figure 1). A total of 68-fold coverage in Pacific Biosciences single-molecule long reads and 91-fold coverage in 10X Genomics read clouds were generated. Primary assembly contigs were scaffolded with chromosome conformation Hi-C data. Manual assembly curation corrected 6 missing/misjoins, reducing the assembly length by 0.8% and the scaffold number by 13.5%.

**Figure 2.**
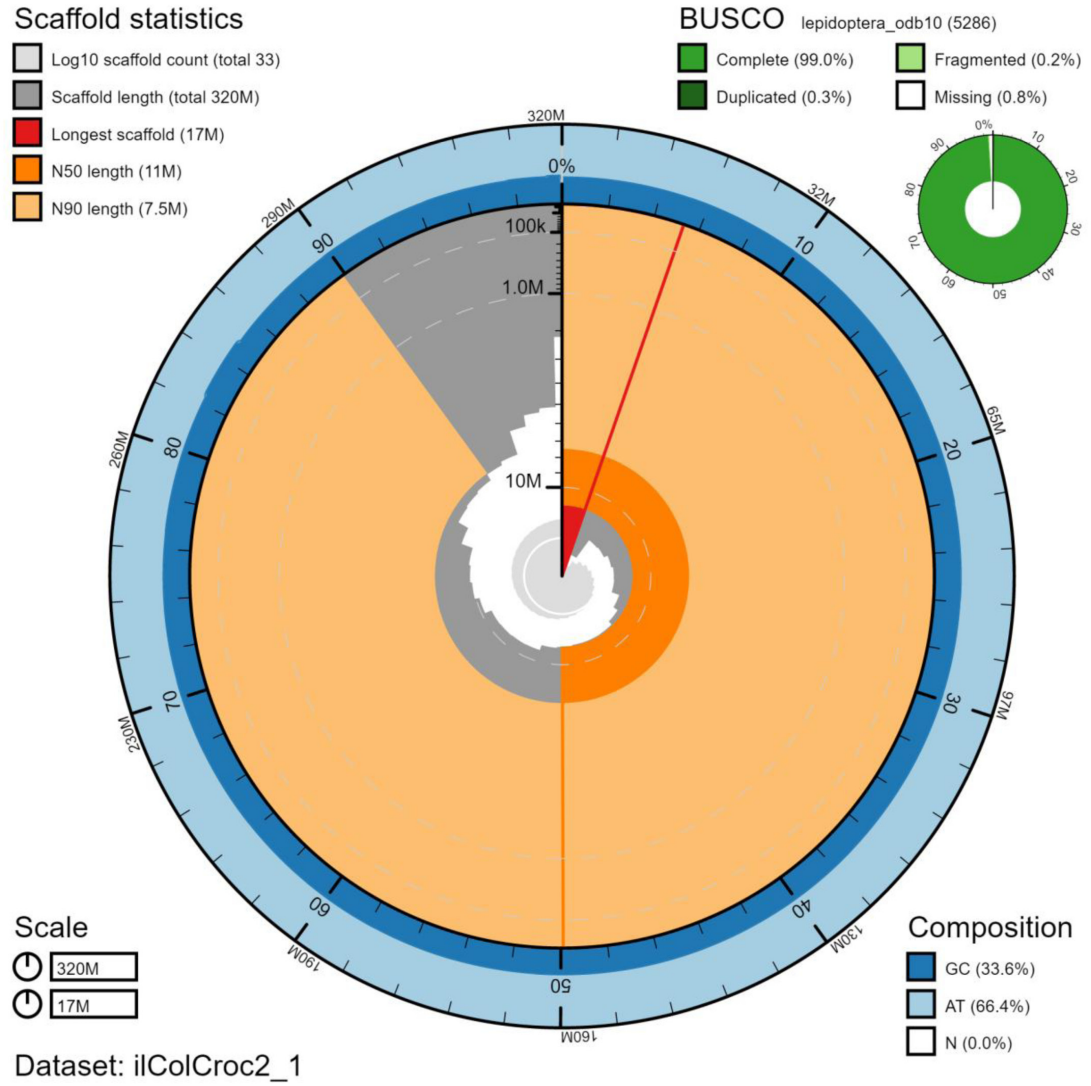
Genome assembly of *Colias crocea*, ilColCroc2.1: metrics. The BlobToolKit Snailplot shows N50 metrics and BUSCO gene completeness. The main plot is divided into 1,000 size-ordered bins around the circumference with each bin representing 0.1% of the 324,912,214 bp assembly. The distribution of chromosome lengths is shown in dark grey with the plot radius scaled to the longest chromosome present in the assembly (17,237,107 bp, shown in red). Orange and pale-orange arcs show the N50 and N90 chromosome lengths (11,204,669 and 7,474,634 bp), respectively. The pale grey spiral shows the cumulative chromosome count on a log scale with white scale lines showing successive orders of magnitude. The blue and pale-blue area around the outside of the plot shows the distribution of GC, AT and N percentages in the same bins as the inner plot. A summary of complete, fragmented, duplicated and missing BUSCO genes in the lepidoptera_odb10 set is shown in the top right. An interactive version of this figure is available at https://blobtoolkit.genomehubs.org/view/ilColCroc2.1/dataset/ilColCroc2_1/snail.

The final assembly has a total length of 325 Mb in 33 sequence scaffolds with a scaffold N50 of 11 Mb ( Table 1). Of the assembly sequence, 100% was assigned to 32 chromosomal-level scaffolds, representing 30 autosomes (numbered by sequence length), and the W and Z sex chromosome ( Figure 2– Figure 5; Table 2). The assembly has a BUSCO ( Simão *et al*., 2015) v5.1.2 completeness of 99.0% (single 98.7%, duplicated 0.3%, fragmented 0.2%, missing 0.8%) using the lepidoptera_odb10 reference set. While not fully phased, the assembly deposited is of one haplotype. Contigs corresponding to the second haplotype have also been deposited.

**Table 1.** Genome data for *Colias crocea*, ilColCroc2.1.

*Project accession data*	
Assembly identifier	ilColCroc2.1
Species	*Colias crocea* (also known as *Colias croceus*)
Specimen	ilColCroc2
NCBI taxonomy ID	NCBI:txid72248
BioProject	PRJEB42878
BioSample ID	SAMEA7523360
Isolate information	Female, abdomen/thorax
*Raw data accessions*	
PacificBiosciences SEQUEL II	ERR6558184
10X Genomics Illumina	ERR6054394-ERR6054397
Hi-C Illumina	ERR6054398
Illumina PolyA RNAseq	ERR6054399
*Genome assembly*	
Assembly accession	GCA_905220415.1
*Accession of alternate haplotype*	GCA_905220445.1
Span (Mb)	325
Number of contigs	42
Contig N50 length (Mb)	11
Number of scaffolds	33
Scaffold N50 length (Mb)	11
Longest scaffold (Mb)	15
BUSCO* genome score	C:99.0%[S:98.6%,D:0.4%],F:0.2%,M:0.8%,n:1658
*Gene annotation*	
Number of protein-coding genes	13,830
Average length of protein coding sequence (bp)	1.631
Average number of exons per gene	8
Average exon size (bp)	359
Average intron size (bp)	2,027

**Figure 3.**
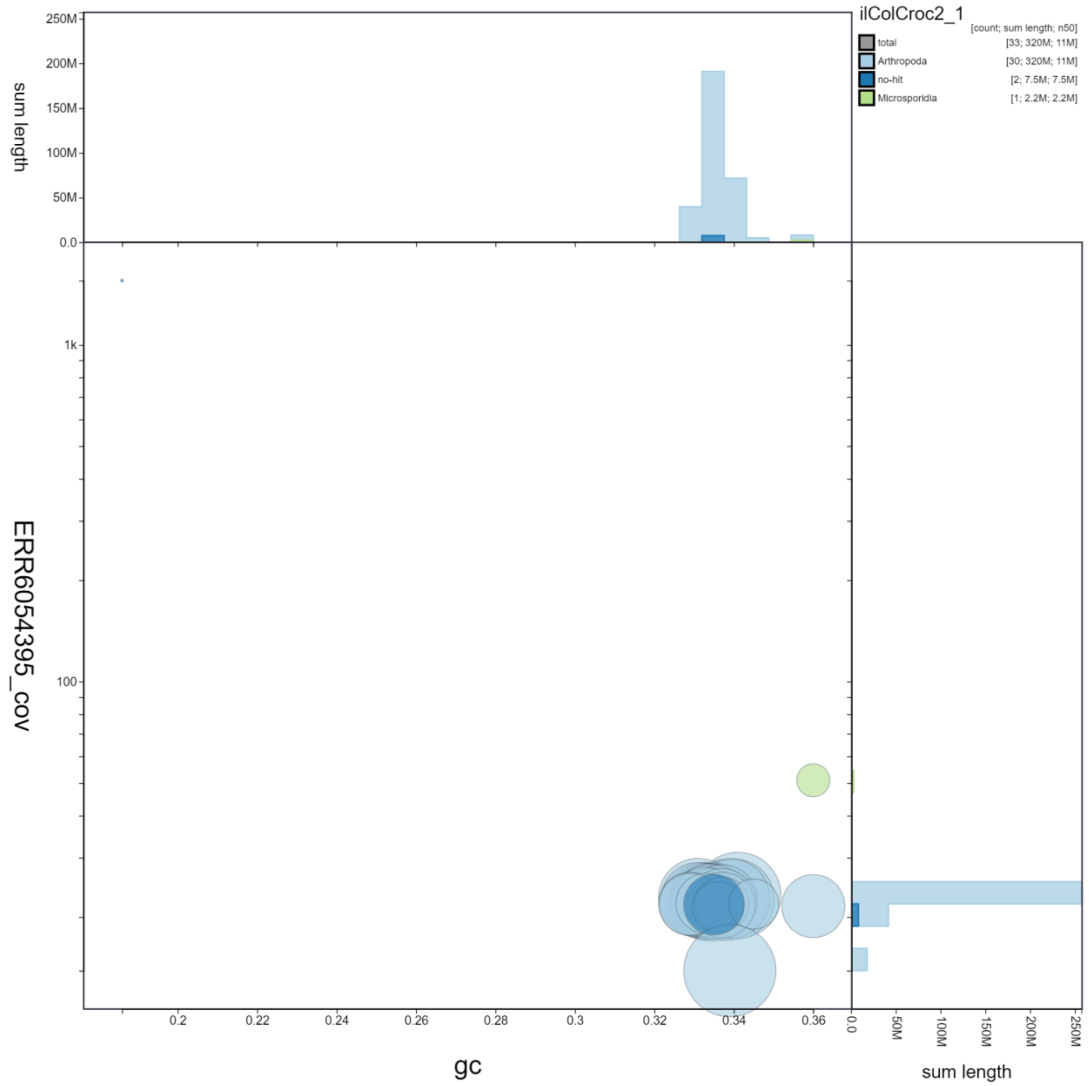
Genome assembly of *Colias crocea*, ilColCroc2.1: GC coverage. BlobToolKit GC-coverage plot. Chromosomes are coloured by phylum. Circles are sized in proportion to scaffold length. Histograms show the distribution of chromosome length sum along each axis. An interactive version of this figure is available at https://blobtoolkit.genomehubs.org/view/ilColCroc2.1/dataset/ilColCroc2_1/blob.

**Figure 4.**
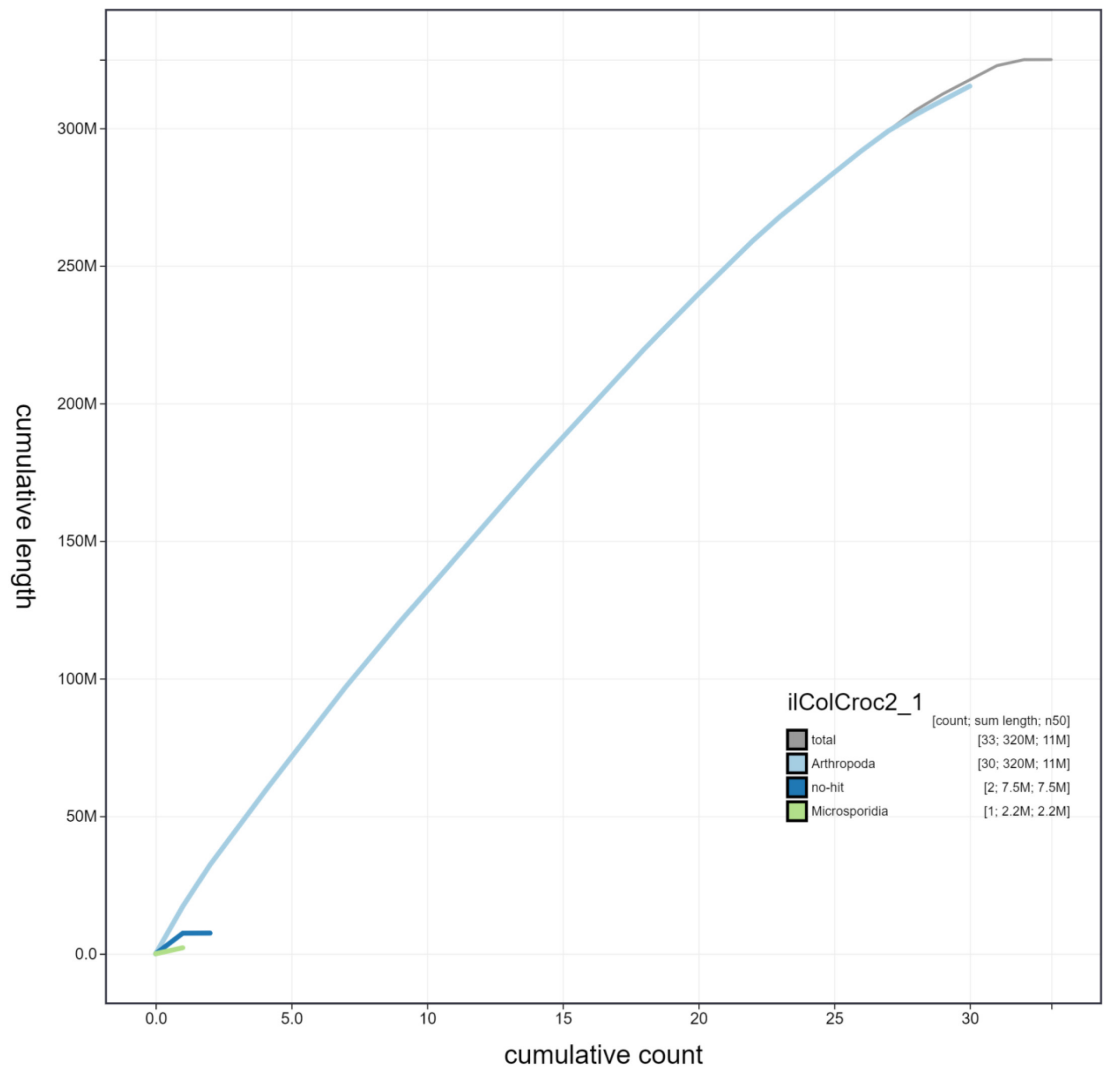
Genome assembly of *Colias crocea*, ilColCroc2.1: cumulative sequence. BlobToolKit cumulative sequence plot. The grey line shows cumulative length for all chromosomes. Coloured lines show cumulative lengths of chromosomes assigned to each phylum using the buscogenes taxrule. An interactive version of this figure is available at https://blobtoolkit.genomehubs.org/view/ilColCroc2.1/dataset/ilColCroc2_1/cumulative.

**Figure 5.**
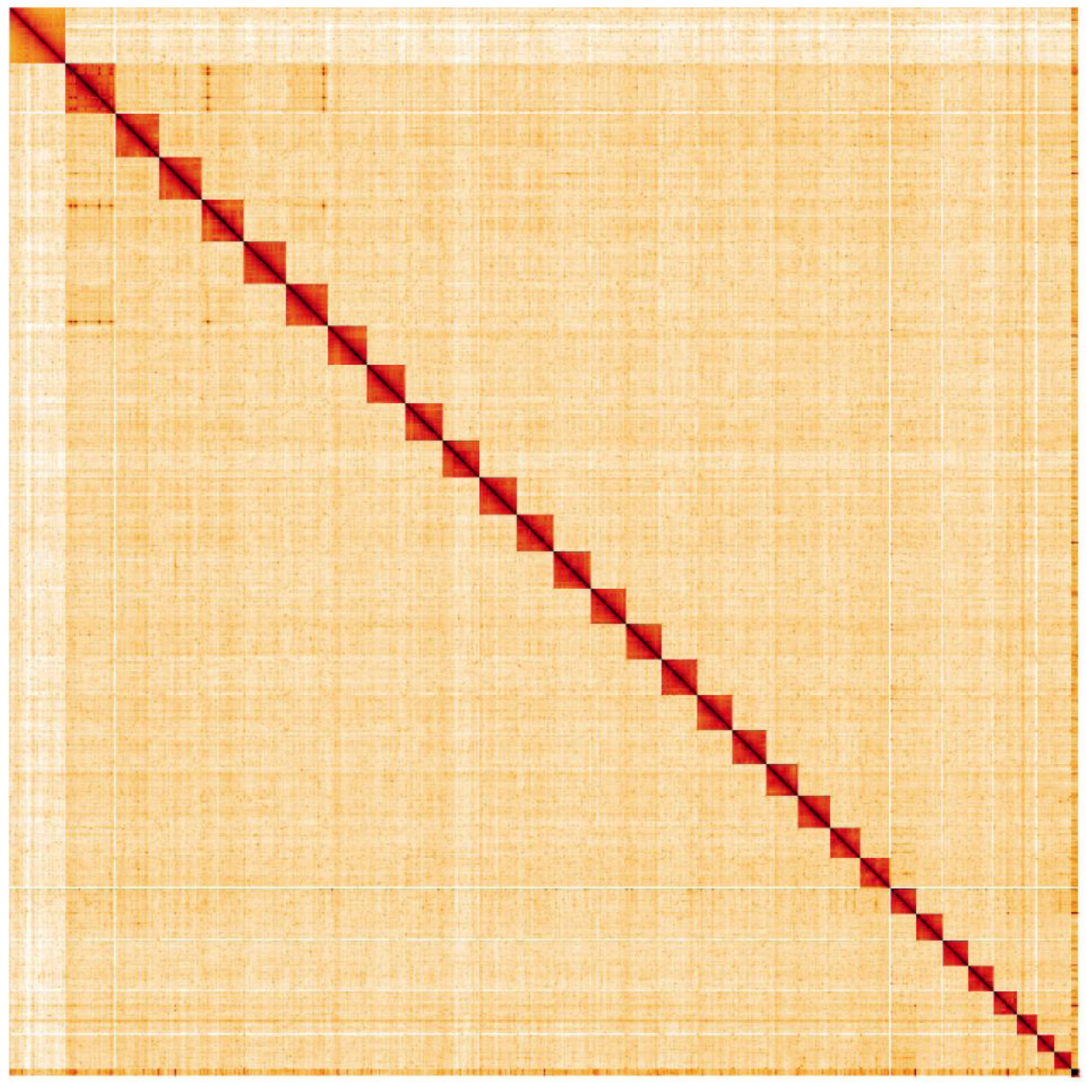
Genome assembly of *Colias crocea*, ilColCroc2.1: Hi-C contact map. Hi-C contact map of the ilColCroc2.1 assembly, visualised in HiGlass.

**Table 2.** Chromosomal pseudomolecules in the genome assembly of *Colias crocea*, ilColCroc2.1.

INSDC accession	Chromosome	Size (Mb)	GC%
HG991959.1	1	15.09	34.1
HG991960.1	2	13.25	33.8
HG991961.1	3	13.17	34
HG991962.1	4	12.85	33.9
HG991963.1	5	12.69	33.4
HG991964.1	6	12.66	33.1
HG991965.1	7	12.04	33.3
HG991966.1	8	11.65	33.2
HG991967.1	9	11.34	33.4
HG991968.1	10	11.32	33.3
HG991969.1	11	11.31	33.4
HG991970.1	12	11.20	33.4
HG991971.1	13	11.13	33.1
HG991972.1	14	10.75	33.3
HG991973.1	15	10.73	33.5
HG991974.1	16	10.69	33.4
HG991975.1	17	10.66	33.6
HG991976.1	18	10.16	33.7
HG991977.1	19	9.83	33.4
HG991978.1	20	9.70	33.3
HG991979.1	21	9.58	33.7
HG991980.1	22	8.79	33.7
HG991981.1	23	8.06	36
HG991982.1	24	7.97	32.9
HG991983.1	25	7.88	32.9
HG991984.1	26	7.47	33.5
HG991985.1	27	7.27	33.3
HG991986.1	28	5.88	33.7
HG991987.1	29	5.28	33.6
HG991988.1	30	5.08	34.5
HG991989.1	W	2.16	36
HG991958.1	Z	17.24	33.9
HG991990.1	MT	0.02	18.7

## Gene annotation

The Ensembl gene annotation system ( Aken *et al*., 2016) was used to generate annotation for the *Colias crocea* assembly ( GCA_905220415.1, Table 1). The annotation was created primarily through alignment of transcriptomic data to the genome, with gap filling via protein to-genome alignments of a select set of proteins from UniProt ( UniProt Consortium, 2019) and OrthoDB ( Kriventseva *et al*., 2008). Prediction tools, CPC2 ( Kang *et al*., 2017) and RNAsamba ( Camargo *et al*., 2020), were used to aid determination of protein coding genes.

## Methods

### Sample acquisition and nucleic acid extraction

A female (ilColCroc2) and a male (ilColCroc3) *C. crocea* were collected from Bujaruelo, Aragon, Spain (latitude 42.7, longitude -0.1) by Sam Ebdon, Alex Macintosh (both University of Edinburgh), Alex Hayward and Karl Wotton (both University of Exeter). Samples were collected using a net and snap-frozen in liquid nitrogen.

DNA was extracted at the Wellcome Sanger Institute (WSI) Scientific Operations core from the thorax of ilColCroc2 using the Qiagen MagAttract HMW DNA kit, according to the manufacturer’s instructions. RNA was extracted from the thorax of ilColCroc3 in the Tree of Life Laboratory at the WSI using TRIzol (Invitrogen), according to the manufacturer’s instructions. RNA was then eluted in 50 μl RNAse-free water and its concentration RNA assessed using a Nanodrop spectrophotometer and Qubit Fluorometer using the Qubit RNA Broad-Range (BR) Assay kit. Analysis of the integrity of the RNA was done using Agilent RNA 6000 Pico Kit and Eukaryotic Total RNA assay.

### Sequencing

Pacific Biosciences HiFi circular consensus and 10X Genomics read cloud sequencing libraries were constructed according to the manufacturers’ instructions. SPoly(A) RNA-Seq libraries were constructed using the NEB Ultra II RNA Library Prep kit. Sequencing was performed by the Scientific Operations core at the Wellcome Sanger Institute on Pacific Biosciences SEQUEL II (HiFi), Illumina HiSeq X (10X) and Illumina HiSeq 4000 (RNA-Seq) instruments. Hi-C data were generated from abdomen tissue of ilColCroc2 using the Arima v2.0 kit and sequenced on HiSeq X.

### Genome assembly

Assembly was carried out with Hifiasm ( Cheng *et al*., 2021). Haplotypic duplication was identified and removed with purge_dups ( Guan *et al*., 2020). One round of polishing was performed by aligning 10X Genomics read data to the assembly with longranger align, calling variants with freebayes ( Garrison & Marth, 2012). The assembly was then scaffolded with Hi-C data ( Rao *et al*., 2014) using SALSA2 ( Ghurye *et al*., 2019). The assembly was checked for contamination and corrected using the gEVAL system ( Chow *et al*., 2016) as described previously ( Howe *et al*., 2021). Manual curation was performed using gEVAL, HiGlass ( Kerpedjiev *et al*., 2018) and Pretext. The mitochondrial genome was assembled using MitoHiFi ( Uliano-Silva *et al*., 2021). The genome was analysed and BUSCO scores generated within the BlobToolKit environment ( Challis *et al*., 2020). Table 3 contains a list of all software tool versions used, where appropriate.

**Table 3.** Software tools used.

Software tool	Version	Source
Hifiasm	0.12	Cheng *et al*., 2021
purge_dups	1.2.3	Guan *et al*., 2020
SALSA2	2.2	Ghurye *et al*., 2019
longranger align	2.2.2	https://support.10xgenomics.com/genome-exome/software/pipelines/latest/advanced/other-pipelines
freebayes	1.3.1-17-gaa2ace8	Garrison & Marth, 2012
MitoHiFi	1.0	https://github.com/marcelauliano/MitoHiFi
gEVAL	N/A	Chow *et al*., 2016
HiGlass	1.11.6	Kerpedjiev *et al*., 2018
PretextView	0.1.x	https://github.com/wtsi-hpag/PretextView
BlobToolKit	2.6.2	Challis *et al*., 2020

### Ethical/compliance issues

The materials that have contributed to this genome note were supplied by a Tree of Life collaborator. The Wellcome Sanger Institute employs a process whereby due diligence is carried out proportionate to the nature of the materials themselves, and the circumstances under which they have been/are to be collected and provided for use. The purpose of this is to address and mitigate any potential legal and/or ethical implications of receipt and use of the materials as part of the research project, and to ensure that in doing so we align with best practice wherever possible.

The overarching areas of consideration are:

- Ethical review of provenance and sourcing of the material;
- Legality of collection, transfer and use (national and international).

Each transfer of samples is undertaken according to a Research Collaboration Agreement or Material Transfer Agreement entered into by the Tree of Life collaborator, Genome Research Limited (operating as the Wellcome Sanger Institute) and in some circumstances other Tree of Life collaborators.

## Data availability

European Nucleotide Archive: Colias croceus (clouded yellow). Accession number PRJEB42949; https://identifiers.org/ena.embl/PRJEB42949.

The genome sequence is released openly for reuse. The *C. crocea* genome sequencing initiative is part of the Darwin Tree of Life (DToL) project. All raw sequence data and the assembly have been deposited in INSDC databases. Raw data and assembly accession identifiers are reported in Table 1.

## Author information

Members of the Darwin Tree of Life Barcoding collective are listed here: https://doi.org/10.5281/zenodo.4893704.

Members of the Wellcome Sanger Institute Tree of Life programme collective are listed here: https://doi.org/10.5281/zenodo.5377053.

Members of Wellcome Sanger Institute Scientific Operations: DNA Pipelines collective are listed here: https://doi.org/10.5281/zenodo.4790456.

Members of the Tree of Life Core Informatics collective are listed here: https://doi.org/10.5281/zenodo.5013542.

Members of the Darwin Tree of Life Consortium are listed here: https://doi.org/10.5281/zenodo.4783559.
