# Supplementary material for: The genome sequence of the clouded yellow, Colias crocea (Geoffroy, 1785)
Source: Wellcome Open Res. 2021 Oct 22;6:284. [Version 1] doi: 10.12688/wellcomeopenres.17292.1 (PMC9490288; doi:10.12688/wellcomeopenres.17292.1)
Supplement: Peer review history

# Peer review: Reviewer response for version 1

**DOI:** 10.21956/wellcomeopenres.19119.r52189

**Published:** 2022-09-20

**Authors:** Rachel A. Steward

**Affiliations:** 1 Department of Zoology, Stockholm University, Stockholm, Sweden

The authors present a highly contiguous and complete genome assembly for the Pierid butterfly, *Colias crocea*. The existence of a female-limited color polymorphism, in which females are either white or orange, has made this and other species in the genus emerging model systems for the study of alternative life history strategies and the maintenance of ancient polymorphisms.

 For this genome assembly, the authors used a combination of sequence data from an orange female, providing detailed information about the sequencing and assembly steps. The authors also used RNAseq data sampled from the thorax of a single adult male to generate an annotation for this assembly, which may limit its scope for describing both the protein coding and noncoding features of this assembly. Overall, I agree with the previous reviewer that a genome assembly of this quality is highly beneficial (and has likely already been useful) for the study of *C. crocea* and for comparative research in butterfly genomics.  

 Critiques:

 I support the critiques of the first reviewer: (1) that more details should be provided about the parameters and options for software used to generate both the assembly and the annotation; and (2) that the manuscript would benefit from a more detailed explanation of the differences between this assembly and existing genomic resources.

 Details are lacking about the annotation. Given the annotation was made using RNA from a single male thorax, it is possible that there were many important coding and noncoding transcripts missing in the sample. The protein prediction tools subsequently used to improve the annotation may have filled these gaps, but I think it is important that the authors both acknowledge these limitations and include some evaluation of annotation quality. The authors could run BUSCO on the protein set or could compare orthologs between this and other well-annotated Lepidopteran gene sets. Also, summary statistics (e.g., average length of protein coding sequence) provided in Table 1 should include some measure of variation (95% CI, etc.)

Are sufficient details of methods and materials provided to allow replication by others?

Partly

Is the rationale for creating the dataset(s) clearly described?

Yes

Are the datasets clearly presented in a useable and accessible format?

Yes

Are the protocols appropriate and is the work technically sound?

Yes

Reviewer Expertise:

Evolutionary ecology, gene expression, alternative splicing

I confirm that I have read this submission and believe that I have an appropriate level of expertise to confirm that it is of an acceptable scientific standard.

# Peer review: Reviewer response for version 1

**DOI:** 10.21956/wellcomeopenres.19119.r46607

**Published:** 2021-11-22

**Authors:** Alyssa Woronik

**Affiliations:** 1 Sacred Heart University, Fairfield, CT, USA

**Summary of the article and contributions:**

 The authors generate a high quality genome for the butterfly *Colias crocea *using PacBio, 10X Genomics, and HiC sequencing data. These sequencing datasets were generated from an orange *C. crocea* female. The resulting genome was annotated using RNA-Seq data from a male *C. crocea. *This is a high quality genome that is more contiguous and complete than a previously published *C. crocea *genome (Woronik *et al*., 2019 ^1^). Because this butterfly species harbors structural variation that results in an alternative life history strategy, such high quality genomes are necessary for in depth study of these (and potentially other) important genomic regions. Overall, this genome will contribute to the understanding of this butterfly’s biology and ecology.

**Critiques: **

1. The authors reported the software and versions used for their assembly. However, I could not find a place where the various options used in those pipelines were stated. Would it be possible for the authors to include the options used in the assembly and annotation commands? This would increase the reproducibility of this work. This could be done by adding another column to Table 3 "Options used" or by including their scripts as a supplemental file.
2. The authors point out that another high quality genome exists for *C. crocea. *This genome was generated from Alba females and annotated using RNA-Seq data from the wings and abdomens of several female pupas. Adding a sentence to the "Background" that highlights the differences between the datasets could be useful for readers interested in using this data as they complement each other nicely.

Are sufficient details of methods and materials provided to allow replication by others?

Partly

Is the rationale for creating the dataset(s) clearly described?

Yes

Are the datasets clearly presented in a useable and accessible format?

Yes

Are the protocols appropriate and is the work technically sound?

Yes

Reviewer Expertise:

Evolutionary genomics and developmental genetics

I confirm that I have read this submission and believe that I have an appropriate level of expertise to confirm that it is of an acceptable scientific standard.

## References

[1] : A transposable element insertion is associated with an alternative life history strategy. *Nature Communications* .2019;10(1) : 10.1038/s41467-019-13596-2 10.1038/s41467-019-13596-2 PMC691773131848330
